# Supplementary figures and images for: Cross-cultural adaptation and validation of a Resistance Training Skill Battery for use in Chinese-speaking adolescents
Source: PeerJ. 2025 Dec 15;13:e20387. doi: 10.7717/peerj.20387 (PMC12713556; doi:10.7717/peerj.20387)

**Appendix 1. Chinese version of Identification of Resistance Training Skills Battery**

**
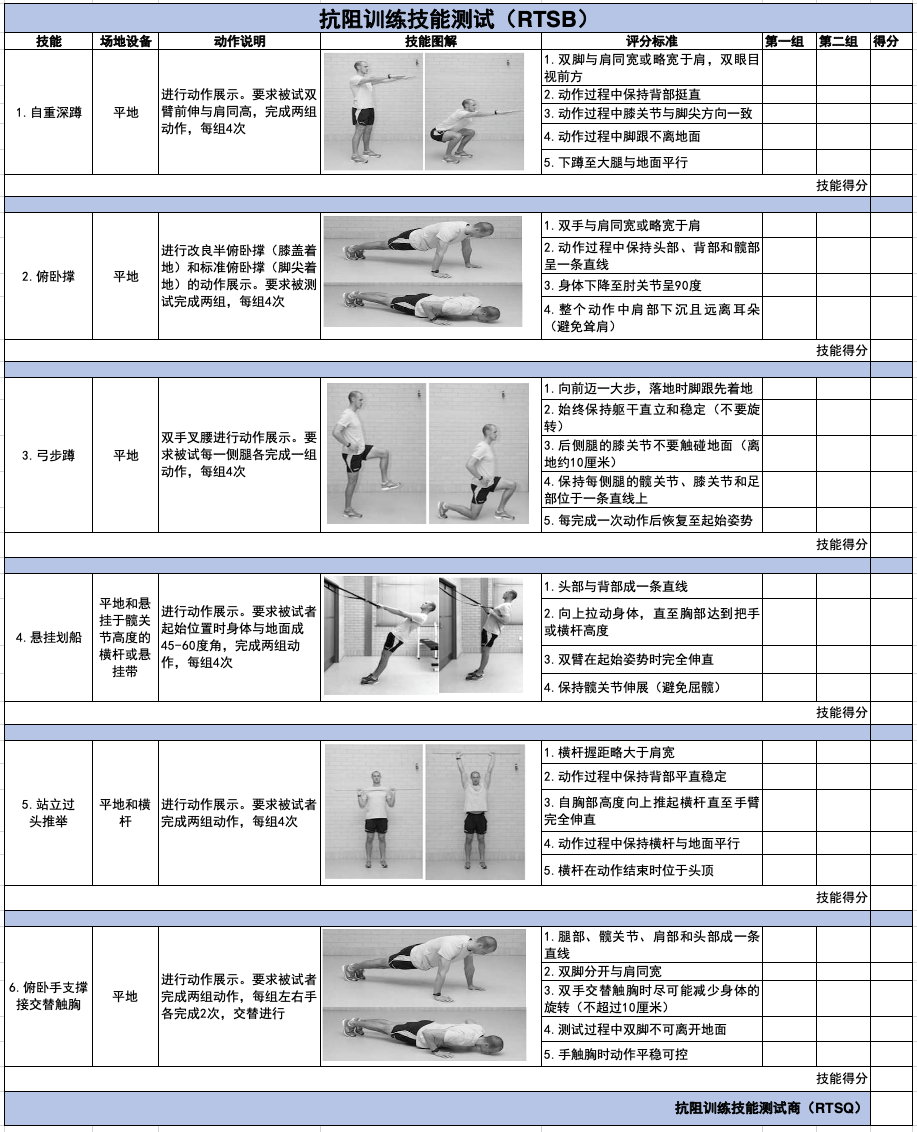
**

Supplement: Supplemental Information 2 [file peerj-13-20387-s002.docx]
