# Supplementary material for: Cross-cultural adaptation and validation of a Resistance Training Skill Battery for use in Chinese-speaking adolescents
Source: PeerJ. 2025 Dec 15;13:e20387. doi: 10.7717/peerj.20387 (PMC12713556; doi:10.7717/peerj.20387)
Supplement: Supplemental Information 3 [file peerj-13-20387-s003.pdf]

Resistance Training Skills Battery (RTSB)

Skill	Materials	Directions	Skill Depiction	Performance Criteria	Set 1	Set 2	Score
1. Body weight squat	Flat surface	Provide a demonstration of the movement. Instruct the participant to perform 4 repetitions of the exercise with the arms extended forward at shoulder height. Repeat a second trial.	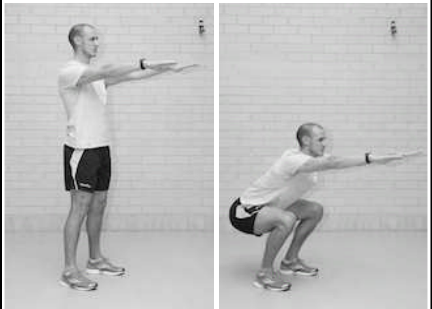	1. Feet are shoulder width or slightly wider apart and facing forward			
				2. Back is kept straight and stable throughout the movement			
				3. Knees point in the same direction as feet during movement			
				4. Heels remain on floor throughout the movement			
				5. Thighs are parallel to the floor at the bottom of the movement			
						**Skill Score**	
2. Push-up	Flat surface	Provide demonstrations of modified (on knees) and full (on toes) push-ups. Instruct the participant to perform 4 modified or full push-ups. Repeat a second trial.	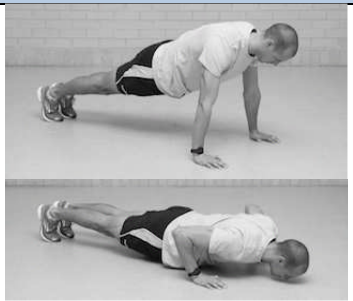	1. Hands are shoulder width or slightly wider apart			
				2. Head, back and hips are held in a straight line throughout the movement			
				3. Body is lowered until elbows are at a 90 degree angle			
				4. Shoulders are held down and away from ears (shoulders are not shrugged)			
						**Skill Score**	
3. Lunge	Flat surface	Provide a demonstration of the movement with hands on hips. Instruct the participant to perform 4 repetitions on the same leg. Second trial is completed with the other leg.	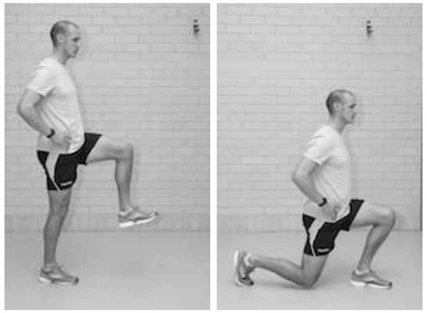	1. Takes an exaggerated step forward and lands heel first			
				2. Torso is kept upright and stable at all times (no twisting)			
				3. Knee of rear leg is almost touching the floor (approx. 10cm)			
				4. There is alignment between hip, knee and foot of each leg			
				5. Returns to starting position in one movement			
						**Skill Score**	

4. Suspended row	Flat surface and bar suspended at hip height or suspension straps with an anchor point	Provide a demonstration of the movement. Instruct the participant to perform 4 repetitions starting with their upper body at a 45-60 degree angle. Repeat a second trial.	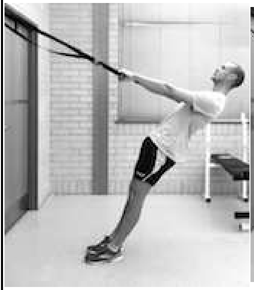 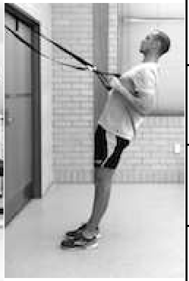	1. Straight line through head and back			
				2. Body is pulled upwards to touch handles or bar at chest height			
				3. Arms are fully extended in the bottom position			
				4. No bending at the hips			
						**Skill Score**	
5. Standing overhead press	Flat surface and barbell	Provide a demonstration of the movement. Instruct the participant to perform 4 repetitions. Repeat a second trial.	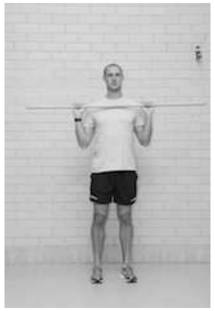 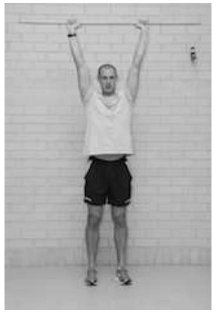	1. Bar is gripped slightly wider than shoulders			
				2. Back is kept straight and stable throughout movement			
				3. Bar starts at chest height and is pressed upward until arms are fully extended			
				4. Bar remains parallel to the ground throughout the movement			
				5. Bar is overhead at the top of the lift			
						**Skill Score**	
6. Front support with chest touches	Flat surface	Provide a demonstration of the movement. Instruct the participant to perform 2 repetitions per side alternating sides each repetition. Repeat a second trial.	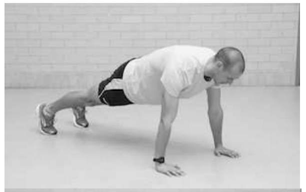 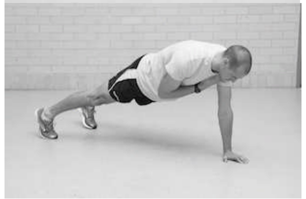	1. Straight line through legs, hips, shoulders and head			
				2. Feet are approximately shoulder width apart			
				3. Minimal rotation of body while changing hand placement (approx. 10cm is acceptable)			
				4. Both feet remain on the ground throughout the entire trial			
				5. Chest touches are performed in a controlled manner			
						**Skill Score**	
						**Resistance Training Skill Quotient**	
